# JBrowse: a dynamic web platform for genome visualization and analysis

**DOI:** 10.1186/s13059-016-0924-1

**Published:** 2016-04-12

**Authors:** Robert Buels, Eric Yao, Colin M. Diesh, Richard D. Hayes, Monica Munoz-Torres, Gregg Helt, David M. Goodstein, Christine G. Elsik, Suzanna E. Lewis, Lincoln Stein, Ian H. Holmes

**Affiliations:** Department of Bioengineering, University of California, Berkeley, California USA; Division of Animal Sciences, University of Missouri, Columbia, Missouri USA; Lawrence Berkeley National Laboratory, Berkeley, California USA; Current affiliation: Genomancer Consulting, Healdsburg, California USA; Ontario Institute of Cancer Research, Toronto, Ontario Canada; US Department of Energy, Joint Genome Institute, Walnut Creek, CA 94598 USA; Department of Molecular Genetics, University of Toronto, Toronto, Ontario Canada

**Keywords:** Genome, Browser, Bioinformatics

## Abstract

**Background:**

JBrowse is a fast and full-featured genome browser built with JavaScript and HTML5. It is easily embedded into websites or apps but can also be served as a standalone web page.

**Results:**

Overall improvements to speed and scalability are accompanied by specific enhancements that support complex interactive queries on large track sets. Analysis functions can readily be added using the plugin framework; most visual aspects of tracks can also be customized, along with clicks, mouseovers, menus, and popup boxes. JBrowse can also be used to browse local annotation files offline and to generate high-resolution figures for publication.

**Conclusions:**

JBrowse is a mature web application suitable for genome visualization and analysis.

## Background

As the web becomes the standard platform for computational genomics, there are clear advantages to performing some analyses on the web client, rather than delegating every task to a central server. Among these advantages are a reduced need for server-hosted resources (with corresponding cost savings) and a faster, more responsive user experience as some latency due to client–server interactions is eliminated.

## Implementation

The JBrowse genome browser leverages these advantages for the visualization of genome annotations. The earliest version of JBrowse was a dynamic HTML application with a JavaScript client, reliant on server-side Bioperl components that were largely inherited from GBrowse [1]. The software was subsequently extended to display next-generation sequencing data [2, 3].

## Results and Discussion

We here report progress in JBrowse robustness, generality, scalability, configurability, and ease of installation. Our goal is not to compare JBrowse with other genome annotation browsers. Many such exist, including JavaScript genome browsers such as Biodalliance [4], pileup.js [5], and Galaxy’s Trackster [6]; other (Common Gateway Interface (CGI)-based) web-facing browsers such as the UCSC Genome Browser [7], Ensembl’s browser [8], and GBrowse [9]; and desktop browsers such as IGV [10] and IGB [11]. A thorough comparison of all these browsers is beyond the scope of the current paper (and should, in any case, be carried out by independent parties for maximal objectivity). Among JBrowse’s strengths (relative to other browsers primarily using dynamic HTML) are its maturity, extensibility (via configuration and callbacks), and emerging ecosystem of plugins and applications. Arguably, its dependencies on older frameworks (dojo, Bioperl) may be considered weaknesses, though JBrowse is an example of how these frameworks may be effectively leveraged. The Bioperl-based indexing tools are, in fact, less central to JBrowse operation than they used to be in earlier versions: indexing is an elective optimization for static site-generation and has been implemented as a proof-of-concept in other languages.

In its current form, JBrowse can be used as a standalone HTML5-based genome browser or embedded in richer web application frameworks. It is highly cross-platform; releases are tested on Mozilla-based browsers (e.g., Firefox), WebKit browsers (e.g., Safari, Chrome) and Microsoft Internet Explorer, and on desktop and mobile platforms (with touchscreen support). JBrowse has, further, been designed to be highly customizable; user interface extensions are straightforward to implement, as are alternative data back-ends. It is also possible to perform analysis tasks directly from within the client.

General improvements to speed and scalability since the last published report include threefold on-disk compression on the server, optimizations to scripts for processing large files, filesystem-oriented optimizations allowing for highly fragmented data (such as draft genomes), and compression of JSON data. The installation process is also streamlined: for example, the setup script installs all dependencies for the (optional) server scripts, so there is no need to install Bioperl separately (and in the desktop application, these dependencies are not required at all; the desktop app is a standalone executable).

JBrowse collects aggregate data on usage statistics by default (this behavior is clearly described in the documentation and can be easily disabled for users sensitive to privacy concerns). From November 2014 to November 2015, our analytics indicate 2671 hosts running JBrowse, of which 705 logged 100 or more access requests during that period. (This is likely an underestimate, since—as noted—users can disable analytics and anecdotal reports suggest that private-sector users frequently do.) The average number of annotation tracks served by these 705 active hosts was 80, with a maximum of 7826. Cumulatively, since analytics data collection began (July 2012), we see 4743 hosts, of which 1168 served 100+ requests. The full JBrowse software package has been downloaded over 100,000 times since July 2012 and viewed by over 64,000 unique nontrivial client addresses, including over 42,000 in the 12 months before November 2015 (we call a web client “nontrivial” if it is running JavaScript, is not pointed at the localhost, and visits the host five or more times).

A few examples of tools that use JBrowse include the web version of Apollo [12], SeqWare [13], DNA subway [14], GenSAS [15], Maker [16, 17], and Afra [18]. Sites that use JBrowse (or JBrowse-based tools) span plant genomics [19–25], animal genomics [12, 26–29], and disease-related databases [30–34].

The rest of this paper reviews various operational aspects of JBrowse, starting with the user interface (UI) and user experience and proceeding to describe configuration: how to hook up different data sources, the various types of track that can be displayed, and the extension of JBrowse via custom code development.

**Table:** 

Usage patterns Running JBrowse with the pre-indexing scripts If JBrowse is to be used as a broad-access viewing portal to a genome annotation database, the greatest efficiency can be gained by using the pre-indexing scripts in the top-level bin/directory. These include prepare-refseqs (for indexing a FASTA file containing the reference sequences and breaking the sequences into manageably-sized chunks), flatfile-to-json (for importing a GFF, BED, or GenBank file as a track), add-bw-track (for importing a BigWig file as a track), and various other utility scripts for adding and removing track configuration stanzas and otherwise managing the track list. Alternatively, the tracks can be loaded directly from a Bioperl-compatible database such as Chado [35] using the biodb-to-json loader script. The final step in this workflow is to build the index of feature and region names using the generate-names script. Running JBrowse without the pre-indexing scripts When standard-format data files are available on the local filesystem (or at a remote URL), a FASTA file can be loaded directly via the Genome menu and annotation tracks can then be created by loading BAM, Wiggle/BigWig, GFF, or VCF files directly via the Track menu. These formats can now be directly consumed by JBrowse (from files or URLs) without the need for JSON indexing; BAM files must, however, be indexed with a BAM index (.bai) file and VCF files with a tabix index (.tbi) file, which may be generated using utilities such as SAMTools [40]. JBrowse uses these index files and HTTP range-request headers to retrieve only the content that it needs to render the particular region in view, allowing very large data files (such as BigWig, BAM, VCF + Tabix, GFF3 + Tabix, or FASTA + faidx) to be browsed quickly over the network in an efficient random-access way. Running JBrowse as part of a larger web application JBrowse can be embedded in a larger web application with its own data sources. The encompassing framework can use the indexing scripts to load data or to directly generate the JSON index files which JBrowse consumes; alternatively, data sources can be provided as a web service (there is a REST API for most features). JBrowse can be started in “embedded mode” (with many user interface features disabled) for compactness. Numerous callbacks are available to implement context-specific actions when the user interacts with the sequence and annotations. Embedding a genome browser in a web application framework has many advantages over a desktop system; for example, users can, in principle, share data and views instantly over the web rather than resorting to sharing screenshots via a side-channel (email). Running JBrowse on the UCSC genome database The ucsc-to-json script can be used to import a UCSC genome database, creating JBrowse index files directly from a UCSC database dump. Demonstration instances for the latest JBrowse release on several genome data sets can be found at http://jbrowse.org/demos. Running JBrowse as a desktop application JBrowse can also be run as a cross-platform desktop application, entirely independently of a web browser, using the Atom Electron framework (atom). The data can be connected to desktop JBrowse in at least two ways: the index files that are generated by the indexing scripts (and normally hosted on the server) can be bundled with the JBrowse application or JBrowse can be used to browse local data files.	

## User interface

**Fig. 1:**
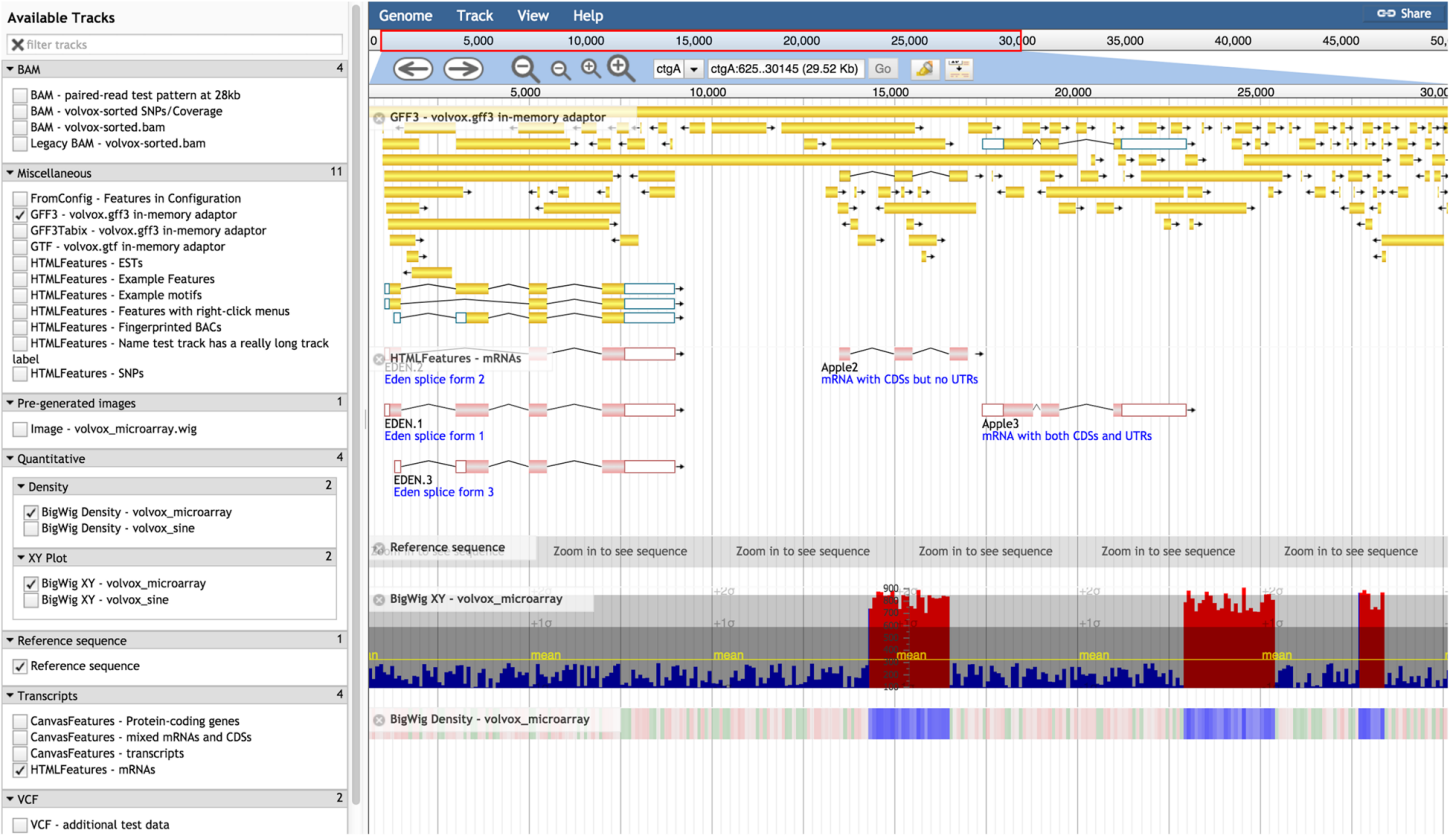
JBrowse screenshot showing (for Volvox test dataset) the hierarchical track selector (*left-hand pane*) and (from *top* to *bottom* in the *right-hand pane*) menu bar, location bar, navigation bar, CanvasFeatures track (with NeatCanvasFeatures plugin), HTMLFeatures track (mRNAs; with NeatHTMLFeatures plugin), Reference Sequence track, Wiggle/XYPlot track, and Wiggle/Density track

**Fig. 2:**
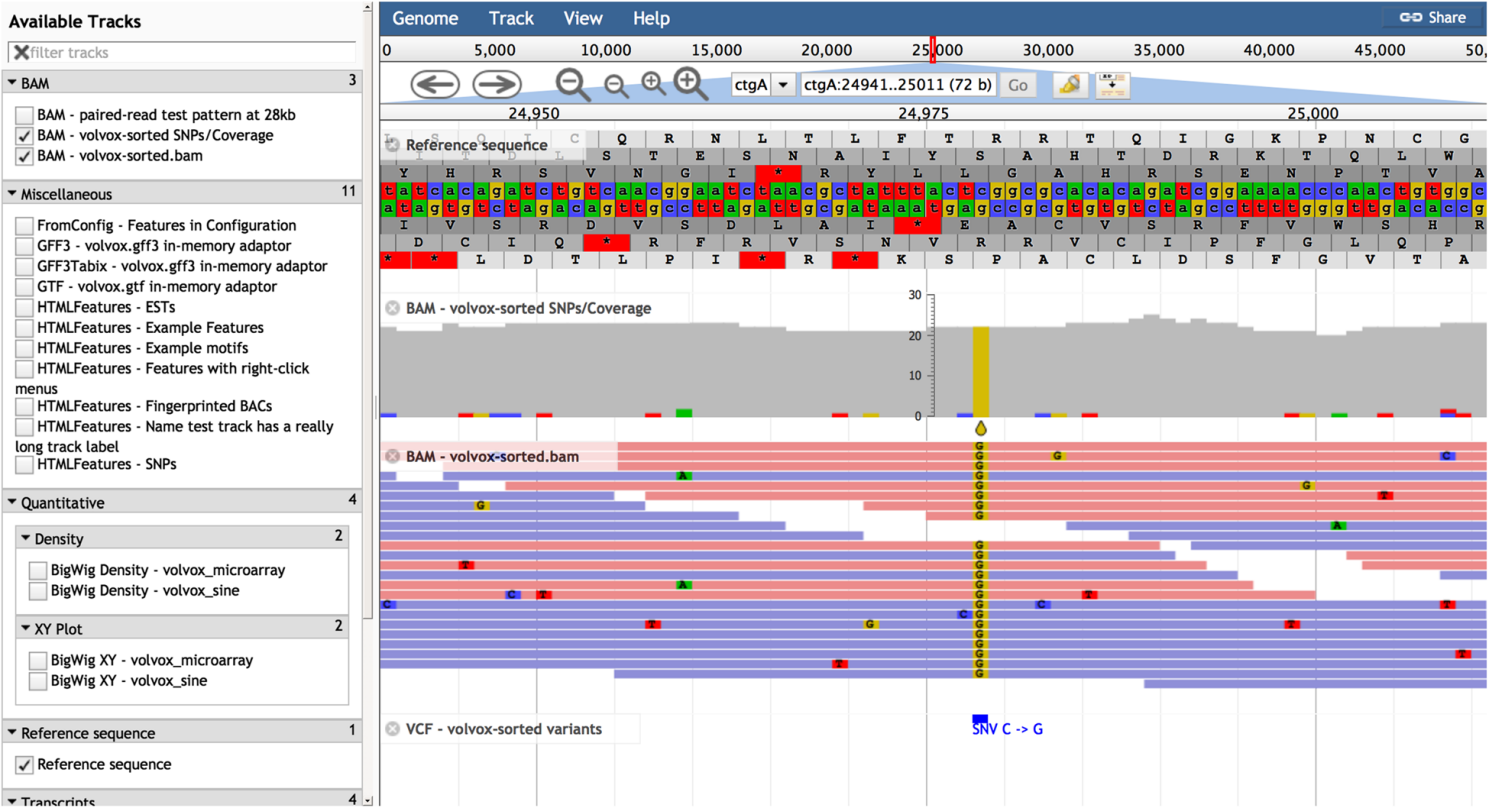
JBrowse screenshot captured with PhantomJS and showing (for Volvox test dataset) the hierarchical track selector (*left-hand pane*) and (from *top* to *bottom* in the *right-hand pane*) Reference Sequence track, SNPCoverage track, Alignments2 track, and HTMLVariants tracks (with NeatHTMLFeatures plugin)

The main view of the user interface is shown in Figs. 1 and 2. At the top is the *menu bar*, which contains the *Genome menu* (allowing the user to select different datasets, each with its own set of reference sequences and annotations), the *Track menu* (where new tracks can be opened from remote or local data sources, or Combination Tracks created from existing tracks), the *View menu* (allowing the highlighting or resizing of quantitative tracks), and the *Help menu*. More detail on Combination Tracks is given below.

Beneath the menu bar is the *navigation bar*, which includes the panning and zooming buttons (and other buttons such as the *highlight button*), the *reference sequence selector* (a dropdown menu which may be configured to sort reference sequences by ascending alphabetical order, descending alphabetical order, or length), the *text navigation box* (enabling navigation to features by name), and an *overview bar* showing the global location of the zoomed-in region.

The tracks themselves are on display in the *genome view*, beneath the navigation bar. Each track also has its own *track menu*, available as a dropdown from the track label. The track menu offers several operations: the user can display track metadata (the “*About this track*” option), pin the track to the top, edit the track configuration directly, export the track data in BED, GFF3 or Wiggle format, delete the track, or set track-specific options (such as changing to a log scale or modifying the height of quantitative tracks or toggling forward/reverse strand display for feature tracks).

Right-clicking on a feature in a track brings up a *context menu* which, by default, has two options: “*View details*” and “*Highlight feature*”. These context menus may be customized to add further options. Left-clicking on the feature goes directly to the “View details” pop-up box, which lists attributes of the feature (and may be further customized).

**Fig. 3:**
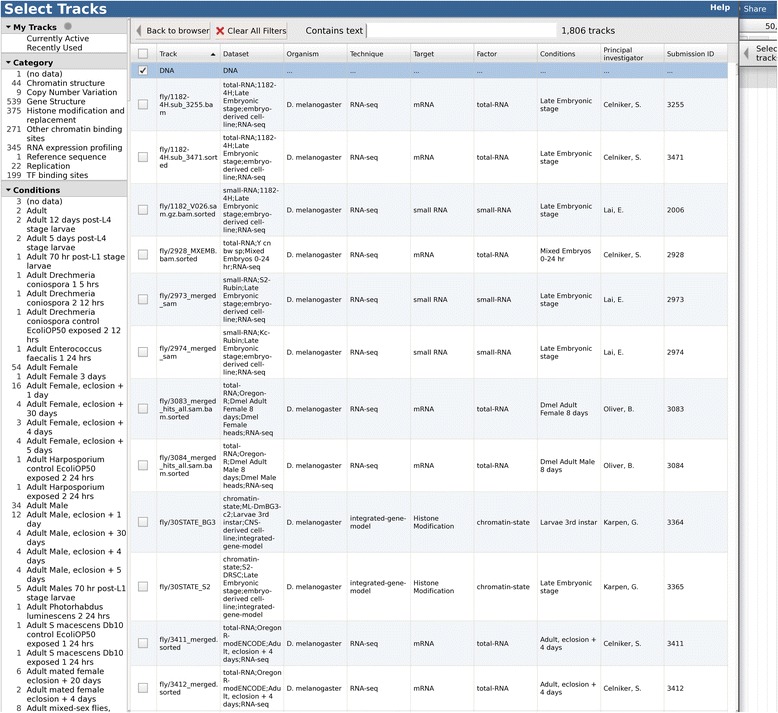
JBrowse screenshot showing large track-set faceted track selector from modENCODE test dataset

The full set of tracks available for potential display is listed in the *track selector* pane, to the left of the genome view. The track selector can be configured to be a simple drag-and-drop list, a hierarchical tree (Figs. 1 and 2), or a “faceted” navigation tool whereby large sets of tracks can be dynamically queried, allowing the user to home in on the track of choice by successively applying filters to the track metadata (Fig. 3). The track selector pane can be resized, or minimized, to allow more space for the genome view.

The *text navigation box* allows the user to navigate directly to particular coordinates or named features of interest. The name index is configurable; multiple aliases for features can be set up. The text navigation box includes an auto-complete feature. In the event that the name search matches multiple features in different locations or on different tracks, a pop-up window allows the user to select the relevant feature.

The *highlight button* allows users to select a region of interest. An internal event is triggered whenever the user highlights a region and this event can be latched onto by plugin extensions; for example, to trigger a sequence homology search of the highlighted region against a database on the server.

The *sequence search track*, created via the Track menu, is an example of a track created by the JBrowse plugin RegexSequenceSearch, which is included with the JBrowse installation by default. This plugin allows the user to search the reference sequence for matches to a query sequence or regular expression, showing the results as a new track.

**Fig. 4:**
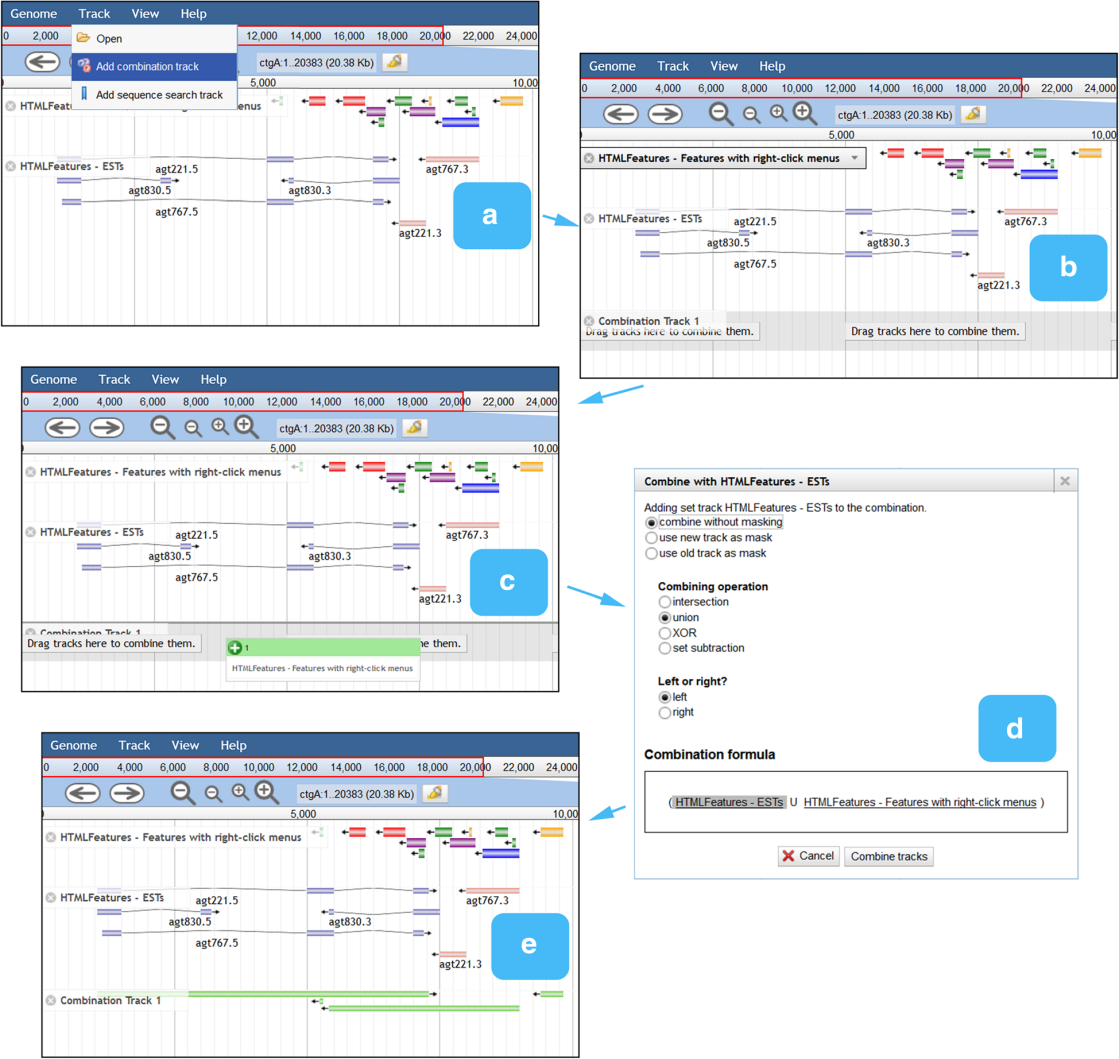
JBrowse “combination track” workflow. Combination tracks are created via the Track menu (**a**), presenting an empty track as a drop target (**b**). Tracks can then be dragged onto the combination track (**c**). The second and subsequent tracks bring up a combination dialog (**d**), with options for set union, intersection, and difference. The results of the set operation are shown in the combination track (**e**)

*Combination tracks*, also created via the Track menu, allow the user to dynamically perform set operations (intersection, union, difference, or exclusive-or) on feature tracks, or arithmetic operations on quantitative tracks. This offers a powerful way to query the track database that parallels the faceted track selector in providing orthogonal filters (Fig. 4). For example, the user could create a sequence search track to find putative TATA boxes and intersect this with a ChIP-Seq track to narrow the search to protein-bound sites.

In addition to the configuration file and the JavaScript API (documented below and in [36]), JBrowse can be controlled in a number of different ways via parameters encoded in the URL string. The most common use of URL parameters is to point JBrowse at a data directory or at a specific feature or region when instantiating the browser, but it also enables several additional features. For example, the track selector, navigation bar, and overview bar can all be disabled in this way, at which point the JBrowse display becomes extremely compact: this is called *embedded mode*. Similarly, the currently visible (and highlighted) regions, and the list of currently visible tracks, can be encoded in the URL string. JBrowse uses this mechanism to implement the *Share* button at the top right of the screen, pressing which generates a “permalink” bookmark for the currently visible location (the same mechanism is also used by the similarly placed *Full-screen view* link in embedded mode to open a new web-browser tab including track selector pane, navigation bar, and overview bar, i.e., to break out of embedded mode). Other extensions available via URL parameters include the import or inline declaration of new features, tracks, or data stores.

The URL-based configuration mechanism also offers an indirect way to generate high-quality figures for publication from the command line using JBrowse. Permalink URLs can be passed to PhantomJS (http://phantomjs.org), a headless client for WebKit (the HTML5 engine underpinning the Chrome and Safari browsers), which can then be used to generate high-resolution PNG, JPEG, or PDF outputs. Fig. 2 in this paper was generated using PhantomJS.

## JBrowse configuration system

When a web browser loads a page containing JBrowse and creates a Browser object (the main controlling object for a JBrowse instance), the first thing the Browser does is to read the configuration information, which can be split across several locations: (1) parameters encoded in the query URL, (2) the configuration JSON object that is passed to the Browser object by the code that creates it, (3) the top-level configuration file(s) in the JBrowse directory, (4) the configuration file(s) in the data directory of the genome being viewed, (5) other configuration files which may be recursively “included” by the above. The JBrowse client merges all the information contained in these configuration files and uses this to decide on (a) the set of available *reference sequences* providing the coordinate system and sequence data for a given dataset (conceptually equivalent to a multiple-sequence FASTA file) and (b) the set of available *annotation tracks* which may be rendered alongside these reference sequences (equivalent, at the data level, to a set of GFF, BED, BAM, Wiggle, and other such annotation files).

Two configuration formats are supported: the first is JSON-based (with file suffix “.json”), the second is a textual format (with file suffix “.conf”). The latter is closely based on the GBrowse configuration format and is easier to edit and maintain by hand than JSON, in particular when specifying JavaScript callback functions in the configuration file. Sites can use either format or a mixture of both. The default shipped configuration of JBrowse uses both: one jbrowse.conf file in the main JBrowse directory for global settings and two files (trackList.json and tracks.conf) in each data directory for dataset-specific configuration.

The capabilities exposed by the configuration file are further discussed in the “JBrowse Configuration Guide” [36].

## Data sources

As noted above (in the “Usage patterns” section), the JBrowse client can obtain its genome annotations from a variety of sources. Data can be loaded directly from GFF3, BAM + BAI, or VCF + TBI flatfiles, which can be hosted on the local filesystem, the web server the client originated from, or an entirely different server. Tracks can also be loaded more efficiently from pre-processed JSON index files (which are hosted on the server and may be generated either on-the-fly or in advance) which support efficient querying of name and coordinate space by employing various data structures, including radix trees and nested containment lists. Annotations can also be loaded via a documented REST API, via SPARQL queries to an RDF data store, or by developing a plugin to implement custom adapters to existing web services.

Using the server-hosted JSON index files (or directly loading server-hosted BAM, VCF, or GFF3 files) is a particularly fast and scalable approach for many applications, requiring very little server compute power beyond the initial pre-processing step. There are several options for pre-generating these indices from data. A suite of scripts is provided to create the JSON files directly from several different sources (including GFF3/BED/GenBank/BAM/FASTA flatfiles, a Chado database, or the UCSC genome database). These scripts are not CGI scripts but pre-indexing tools; they only need to be run once (or every time a new track is added). Used in this way, the JBrowse software can be considered a “static site generator”. As noted in the preceding paragraph, in many cases (for example, BAM files) it is not necessary to use JBrowse’s JSON indices; standard tools such as SAMTools and Tabix can be used to generate index files. Alternatively, developers can readily write code in other languages to generate the indices dynamically at runtime, using the documented REST API and the associated JSON schema.

The JSON-based file format (along with the other, more standard file formats used by JBrowse, such as GFF3, VCF, and BAM) is stable and consistent between JBrowse sites, so that it is straightforward for other JBrowse instances (or indeed third-party browsers) to access the same data via the REST API. There are also various options via which a JBrowse instance can combine data from multiple sites, assuming that the site’s Cross Origin Resource Sharing (CORS) policy is appropriately configured. These options can include fetching BAM, VCF, or GFF3 files from other sites; accessing external REST APIs such as myvariant.info; or integration with platforms like CoGe or Araport, as implemented by iPlant [21].

To allow for large sets of annotation tracks which may be subdivided according to multiple, quasi-orthogonal classification schemes—for example, in genome projects such as modENCODE [37, 38] or ENCODE [39] where a large number of experiments have been performed by various laboratories, on various cell lines, using various protocols—JBrowse supports *faceted navigation* of track lists, whereby the relevant tracks can be selected by dynamically applying a series of intersecting filters within the track selector. In order to support this, track metadata (including the “facets”, i.e., searchable fields) can be specified directly in the configuration file, in a separate CSV-format file (comma-separated value) whose location is specified in the main configuration file, or in a custom metadata store.

The collection of a set of reference sequences, plus all tracks using those reference sequences as a coordinate system, is referred to as a *dataset*. A dataset must be initialized either by indexing the reference sequences on the server (e.g., using the supplied script prepare-refseqs, which breaks the reference sequences into chunks of manageable size) or by loading a FASTA file directly into the browser client. Multiple datasets can be specified in the configuration file; they are then presented within a single JBrowse instance via the Genome menu.

A particular challenge to scalability is presented by genomes in “draft” form, which may be fragmented into thousands or tens of thousands of contigs, each of which is a reference sequence. The drop-down menu of reference sequences can be sorted by length or by another custom sort order, but in this scenario must typically be augmented by other discovery mechanisms. It is recommended that the administrator also configure the name index (to which reference sequence names are in any case automatically added) and potentially the category mechanism for the faceted track browser. Since it is also straightforward to craft URLs that link directly to features, the administrator can additionally configure a BLAST server or other such tool to allow users to discover features by sequence homology search.

## Track types

The core visual elements of a JBrowse track are sequence data, feature glyphs, and quantitative data. Although most tracks predominantly feature one of these elements, the elements are often also combined, composed, or staged together to visually highlight aspects of the data. For example, the SNPCoverage track, visualizing next-generation sequence reads aligned to the reference sequence, condenses reads into a histogram of coverage density and highlights potential single-nucleotide variants as colored characters.

### Sequence (FASTA) tracks

The Sequence track displays forward and reverse strands of the reference sequences and six conceptual translation frames (a non-standard codon translation table can be specified at configuration). JBrowse can load sequence data from FASTA files, indexed FASTA files, and pre-processed sequence data converted into JSON files. It can also display data from a file simply containing chromosome names and sizes and with no sequence data, in which case no actual sequence track is used.

### Feature (GFF, BED, GenBank) tracks

The two types of tracks currently available for displaying annotations from GFF or BED files are HTMLFeatures and CanvasFeatures, each of which has relative advantages in various situations. (An experimental third type of feature track, NeatCanvasFeatures, which extends CanvasFeatures with specialized intron cartoons, is described in the section "Writing plugins"). These tracks can display features with optional structured subfeatures (as, for example, in a GFF3 file) and so are ideal for displaying gene models (with component exons, introns, UTRs), transcript alignments, single-nucleotide polymorphisms (SNPs), transposons, repeats, and so on.

The HTMLFeatures track uses elements of the HTML Domain Object Model (DOM), such as DIV elements, to build up the displayed features. Configuration options allow the customization of many aspects of the track, including the layout parameters, density, height, text description, and Cascading Style Sheets (CSS) classes for various components. Around 22 CSS feature glyphs are included with JBrowse by default. The inbuilt documentation describes how to configure an HTMLFeatures track to use these CSS glyphs with various types of feature, including “two-level” features where a parent feature uses one CSS glyph and the child features use a different glyph.

The CanvasFeatures track paints features directly onto an HTML Canvas element. Early versions of JBrowse were designed not to rely on this element, but the Canvas element has been supported by all major web browsers since 2009 and is now available to 97 % of web users (according to https://www.w3counter.com/), 99 % of jbrowse.org visitors, and 99.9 % of JBrowse users.

As with HTMLFeatures, CanvasFeatures comes with a number of pre-built “glyphs” with which different features can be associated, and these glyph–feature associations can be specified (along with various other layout, rendering, and general visualization options) in the configuration file. Unlike HTMLFeatures, however, the CanvasFeatures glyphs are not restricted to being based on HTML elements and so a more expressive visual language is available. It is also straightforward to extend the glyphs by writing new glyph classes. Like GBrowse glyphs [9], CanvasFeatures glyphs are modular and can comprise subfeatures. To create an entirely new glyph visualization requires only a small amount of JavaScript.

As noted above, each track has relative advantages. CanvasFeatures tracks are generally easier to configure and faster to render than HTMLFeatures tracks; and, as described above, it is easier to develop custom CanvasFeatures glyphs. On the other hand, HTMLFeatures makes certain UI customizations and extensions easier, since it provides a series of ready-made DOM objects with callback hooks for events like “click” and “drag” that the developer can latch onto. The Apollo annotation plugin for JBrowse uses HTMLFeatures tracks to enable drag-and-drop functionality. The NeatHTMLFeatures and NeatCanvasFeatures plugins, described in the “Customization” section, illustrates implementations of a custom intron rendering using both types of track.

### Quantitative (Wiggle, BigWig) tracks

Numerical data associated with intervals or individual nucleotides, as stored in Wiggle and BigWig files, can be plotted using histograms (the Wiggle/XYPlot track) or heat maps (the Wiggle/Density track). Mousing over either kind of track brings up a cursor and popup text displaying (as a numeric value) the data point currently under the mouse pointer.

JBrowse can load quantitative data directly from BigWig files stored on the server, with no need for preprocessing. Both kinds of Wiggle track can be extensively configured to customize coloring, thresholding, scaling, cutoff behavior, global error bars, and other styling.

### Alignment (BAM) tracks

Three types of track are available for rendering the data in BAM files (reference-aligned reads): Alignments (a highly configurable track with customizable click behavior which renders reads as individual HTML elements), Alignments2 (a faster track, optimized for deep-coverage datasets, which renders reads directly onto an HTML Canvas element), and SNPCoverage (a track which dynamically calculates and visually highlights SNPs from BAM data, including nucleotide frequencies).

For deep-coverage BAM files, Alignments2 is recommended for performance reasons (due to Alignments having a longer rendering time and SNPCoverage performing a large number of calculations in order to compute SNPs on-the-fly). It is possible to convey the same information as an SNPCoverage track by pre-computing a BigWig file of the coverage and a VCF file of putative SNPs, both of which can then be displayed with other track types (e.g., Wiggle/XYPlot and VCF tracks, respectively).

BAM files used with JBrowse must be compressed and coordinate-sorted. A wide array of options are offered for customizing details of BAM track operations, including handling of duplicate, multiply mapped and paired-end reads, calculation of coverage histograms, visualization of mismatches and SNPs, and track size, coloring, and styling.

### Variant (VCF) tracks

The HTMLVariants and CanvasVariants track classes are derivatives of HTMLFeatures and CanvasFeatures that are optimized for displaying the potential large amounts of detailed data that go along with each variant. JBrowse can serve these tracks directly from VCF files; however, the VCF files must be compressed with bgzip and indexed with tabix, both of which are available as part of the SAMtools package [40] and/or the related HTSlib package.

### Image tracks

JBrowse can load images directly from the server and display them aligned to the genome, using the FixedImage track. Earlier versions of JBrowse used pre-rendered images to display features and histograms. This is no longer the case (JBrowse now includes code to render these directly from the client), but JBrowse has retained the image track API to support track images rendered on the server.

### Miscellaneous derived tracks

NeatCanvasFeatures is a specialized track which extends CanvasFeatures with “hat” cartoons for introns. It is described in the "Writing plugins" section.

The FeatureCoverage track is a special subclass of Wiggle/XYPlot that dynamically computes and displays coverage for a feature file (e.g., a BAM file).

The base-pair track is an example FixedImage track wherein base-pairing arcs (indicating secondary structure of the reference sequence) are pre-rendered, on the server, using the GD library.

## Customization

JBrowse is designed to be straightforward to embed, customize, and extend. Relevant design features include the event framework, the plugin mechanism, and many callback hooks which can be overridden to change or augment the default behavior of various UI elements.

The majority of JBrowse code is contained in the JavaScript part of the software repository and is organized according to the MVCS (Model-View-Controller-Store) pattern [41]. The client-side framework used is Dojo [42]: view components make use of Dojo’s Dijit widgets, while modules are defined using Dojo’s AMD (Asynchronous Module Definition) format. The code base can be extended by placing new source files directly into the directory tree, by adding plugin extensions, or by defining new functions in the configuration files.

As a general code convention, the names of abstract classes, abstract mixins, private variables, and private methods are preceded with a leading underscore.

### Initialization milestones

The phases of startup are synchronized using named promises called *milestones* (implemented as dojo/Deferred events), which can be hooked onto by plugins and other JavaScript code using the “afterMilestone” method.

Milestone events are shown in Table 1. Each milestone may be attached to callback functions that will be executed when the corresponding milestone completes.

**Table 1:** JBrowse initialization milestones

Milestone name	Milestone is reached when…
initPlugins	…all plugins (but not feature tracks and stores) have been initialized
loadRefSeqs	…all reference sequences have been loaded
loadUserCSS	…the user-defined style sheet has been loaded
loadNames	…the feature search names, used by the text navigation box and autocomplete, have been loaded
initView	…the GenomeView object and menus have been created
initTrackMetadata	…the track metadata has been loaded
loadConfig	…the configuration file(s) have been successfully parsed
createTrack	…the track selector has been initialized
completely initialized (note the space)	…all major services have completed initialization

### Sequence feature models and stores

Classes modeling individual sequence features conform to the *Feature API* (exemplified by and documented in the source for the class JBrowse/Model/SimpleFeature) by providing accessor methods for various feature attributes (start and endpoint, ID field, tags, score, parent and child relationships for modeling super- and sub-features), some of which are mandatory for the various different types of track.

By contrast, classes modeling *sources* of sequences and sequence features generally inherit from JBrowse/Store/SeqFeature and implement the *Feature Store API*, including methods for retrieval of global and local statistics, sequence, and feature data.

Typically, different Feature Stores will provide their own custom implementations of the Feature API. More information on the Feature API and Feature Store API, and other useful classes, can be found in the “JBrowse Configuration Guide” at GMOD [36].

### Callback hooks

JBrowse feature tracks, and individual JBrowse features, can be customized using JavaScript functions added by the developer. These functions are called every time a feature in a track is drawn, allowing customization of virtually anything about the feature's display. Since all of the feature's data and attributes are accessible to the customization function, an individual feature’s appearance can be customized based on these data.

#### Custom creation and post-processing of features in HTMLFeatures tracks

The HTMLFeatures track offers two callback functions that can be overridden (via the config file) to control the way that individual features are rendered:

- The 'hooks->create' callback can be used to control what DOM object is created to represent an individual feature. It is called with track and feature objects as arguments. The default just creates a DIV element.
- The 'hooks->modify' callback can be used to modify the DOM object for a feature, *after* it is created. The function is called with track, feature, and DOM element objects as arguments.

#### Custom rendering and styling of glyphs in CanvasFeatures

Unlike HTMLFeatures tracks, canvas-based feature tracks don't use modify and create hooks. Instead, any of the style attributes (controlling color, labels, dimensions, and other aspects of the appearance and behavior of the glyph) can be specified as dynamic functions in the configuration file rather than static values. As an example of how this can be applied, the “JBrowse Configuration Guide” gives a config file snippet that can be used to color homozygous and heterozygous variants differently in a CanvasVariants track (which inherits from CanvasFeatures; see the “Variant (VCF) tracks” section).

#### Custom “View details” pop-ups

By default, the “View details” pop-up box (which can be selected by either left-clicking on a feature or right-clicking then selecting “View details” from the context menu that appears) displays a simple report about the genome feature that was clicked, including primary data (name, location, reference sequence), sub-feature structure, and additional miscellaneous attributes from the source annotation file in a tabular name-value format. Callbacks can be added to dynamically change the names, values, and mouseover behavior of these extra miscellaneous attributes or to remove them from the “View details” pop-up altogether.

#### Custom mouseover and click behavior, context menu options, and “About this track” pop-ups

Most of the interactive behavior of a feature track can be customized by attaching functions to various callback hooks. This process is described in detail in the configuration guide, illustrated by numerous code snippets. The customizations can include the behavior when a feature is moused-over or left-clicked, the options in the context menu that appear when it is right-clicked, or the “About this track” popup (which displays track metadata analogously to the “View details” popup’s display of feature attributes and can be customized in similar ways).

### Publishing and subscribing to client events

JBrowse client events are implemented using the dojo/topic message bus from the Dojo library. Extensions can subscribe to particular events in order to be notified when certain UI changes happen (for example, highlighting a region generates an event, which can be latched onto with a callback that triggers a request for the server to BLAST that region against a database). In select cases, extensions can also publish events as a way of forcing the UI into certain states or transitions (for example, events can be used in this way to force the browser to load a new track in response to some other circumstance or notification).

Events are documented in the “JBrowse Configuration Guide”. The DebugEvents plugin logs events to the console, and can be used to monitor events as they are triggered by user interactions with the browser.

### Writing plugins

An extension to JBrowse is typically composed of several parts: JavaScript classes for initialization, View and Store code, CSS files for styling UI components, image files for buttons, and other visual components. The JBrowse plugin mechanism allows these files to be collected in one central place and to be automatically loaded when JBrowse is launched. Each plugin is given its own namespace so that any track types, feature glyphs, track selector, or parser/store classes can be easily specified in config files by just referencing their path (for example, MyPlugin/View/Track/CustomTrack can be specified in the track configuration file to refer to a new custom track type).

Each plugin is placed in its own subdirectory of the top-level ‘plugins’ directory of the JBrowse installation (for example, plugins/MyPlugin) and should have subdirectories named ‘js’, ‘img’, and ‘css’ for, respectively, code, images, and stylesheets. The plugin must also be declared in the configuration (typically in the top-level JBrowse configuration file). JBrowse loads the plugin using the Dojo AMD mechanism: an AMD module definition file is required (‘plugins/MyPlugin/js/main.js’). A stylesheet will also be automatically loaded if it is present (‘plugins/MyPlugin/css/main.css’). A script is provided with JBrowse to create this skeletal file and directory structure, automating the first step of developing a new plugin.

Several plugins are provided with JBrowse and are listed in Table 2. The source code for these plugins has been commented to assist developers of new plugins.

**Table 2:** JBrowse plugins

Plugin name	Plugin description
RegexSequenceSearch	Allows the user to enter a simple sequence motif of regular expression, then creates a new feature track showing matches to this motif. Illustrates implementation of JBrowse menu extensions and popups (with CSS), creation of a new track, access of the reference sequence, and dynamic creation of features based on client-side computation
NeatHTMLFeatures	Extends HTMLFeatures tracks to render introns using the standard cartoon representation of a “hat”-shaped elbow line and features with gradient “tube” effect. Illustrates the implementation of custom glyphs. Draws the intron using Scalable Vector Graphics (SVG)
NeatCanvasFeatures	The analog of NeatHTMLFeatures for CanvasFeatures tracks
HideTrackLabels	Extends the toolbar to implement a new button that hides track labels. Illustrates the implementation of toolbar buttons
DebugEvents	Logs all milestone and globally published events to the browser’s JavaScript console. Illustrates the use of plugins for debugging and subscription to dojo/topic events
CategoryUrl	Adds a new ‘category’ URL parameter that displays all tracks in a particular category. Illustrates the implementation of new URL parameters
PubAnnotation	Allows JBrowse to display text annotations from PubAnnotation. Illustrates the creation of a new feature store that queries an external repository

The plugins in this table are included in the JBrowse source distribution, in many cases for expository purposes. Other plugins may readily be developed and added to any given JBrowse installation

### Writing new back-end data services: REST and JSON APIs

The current version of JBrowse does not require a dynamic server back-end (only run-once scripts that optionally can be used to generate the JSON index files) but it is designed to be easy to integrate with other server-based services. In particular, the JBrowse data interface includes REST adaptors that can readily connect to web-facing stores of features (GFF-, BED-, or BAM-style) and quantitative scores (Wiggle-style). The back-end store needs to implement a simple RESTful API that serves statistics on the number and density of features (and/or the range of scores) on a reference sequence and, optionally, on a sub-interval of that sequence or a sub-division into bins. The store also needs to implement a local query method. The text search database (searching and autocompleting feature names) and the reference sequence store can also be connected via RESTful interfaces. Finally, the JSON and text formats for configuration, track lists, track metadata, and the tracks themselves can be dynamically generated by server code (as opposed to being pre-generated by the indexing scripts).

### Future plans

We are currently developing a registry of JBrowse plugins (including third-party extensions) and another registry of publicly accessible data instances, both of which will be hosted on http://jbrowse.org. Announcements and discussion of planned developments for JBrowse will be available on the project blog on http://jbrowse.org. The project mailing lists, which often include announcements, are also linked from http://jbrowse.org.

## Conclusions

JBrowse is a fully-featured genome browser that is capable of visualizing diverse types of genome-located data, located in a variety of different data stores, and of interfacing to other client and server applications.

### Availability of data and materials

All source code is available from the git repository, linked to from http://jbrowse.org, under a choice of Open Source Initiative-compatible licenses, including the GNU Lesser General Public License and the Artistic License. A Docker image that can be used to launch JBrowse containers is also available from http://jbrowse.org.
